# Correction: Full length RTN3 regulates turnover of tubular endoplasmic reticulum via selective autophagy

**DOI:** 10.7554/eLife.107151

**Published:** 2025-04-15

**Authors:** Paolo Grumati, Giulio Morozzi, Soraya Hölper, Muriel Mari, Marie-Lena IE Harwardt, Riqiang Yan, Stefan Müller, Fulvio Reggiori, Mike Heilemann, Ivan Dikic

**Keywords:** None

Grumati P, Morozzi G, Hölper S, Mari M, Harwardt M-LIE, Yan R, Müller S, Reggiori F, Heilemann M, Dikic I. 2017. Full length RTN3 regulates turnover of tubular endoplasmic reticulum via selective autophagy. *eLife* **6**:e25555. doi: 10.7554/eLife.25555.

Published 15 June 2017

We were notified, via PubPeer, of an error in Figure 4. In panel 4B Wild-Type column, the Vinculin WB (our gel loading control) was duplicated resulting identical to the Vinculin WB (our gel loading control) in panel 4A Atg5–/– column. We predict that these duplications occurred while we were assembling the figure. We used Vinculin as loading control of the western blot; therefore, this mistake does not have impact on the interpretation or integrity of the data.

While correcting this mistake, we also noticed that, in panel 4A Atg5–/– column, we reported the original, uncropped, western blot for RTN1A. We edited the same western blot imagine making it uniform with the others. The western blot is the same, we did not charge the original one.

No text corrections are needed, these mistakes are not affecting the results reported in the manuscript.

The corrected Figure 4A and 4B are shown here:

**Figure 1:**
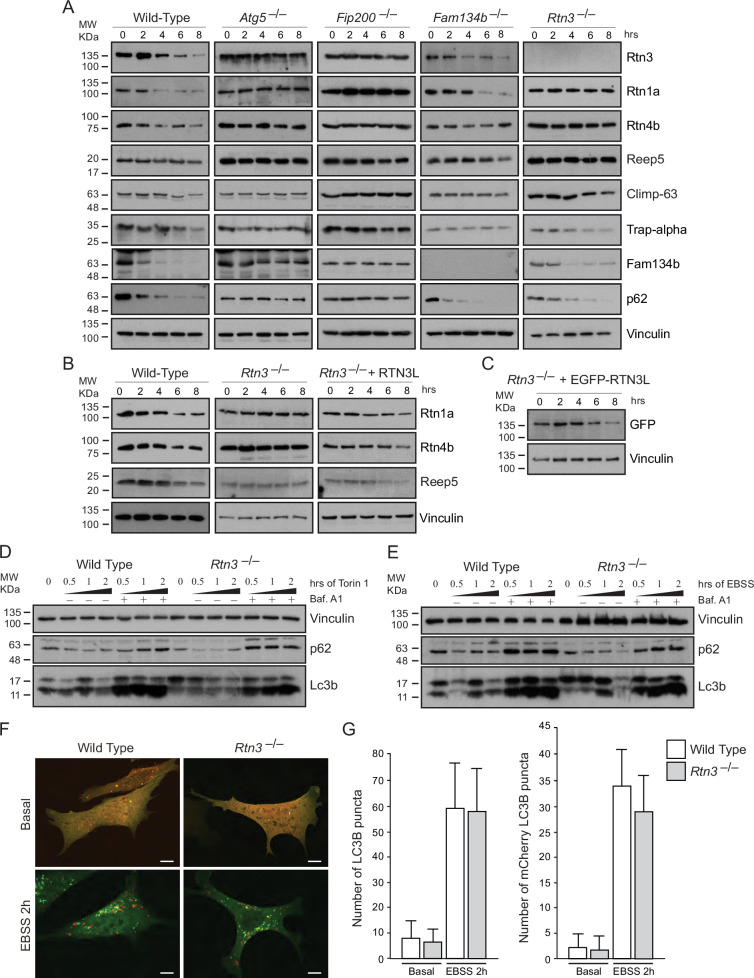


The originally published Figure 4 is shown for reference:

**Figure 2:**
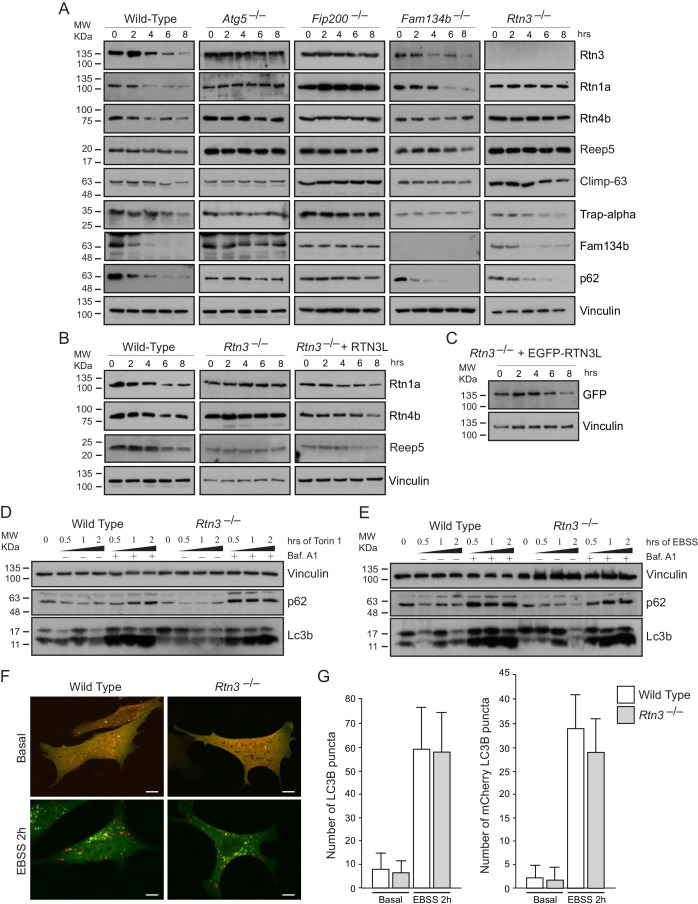


The article has been corrected accordingly.

